# National physical activity and sedentary behaviour policies in 76 countries: availability, comprehensiveness, implementation, and effectiveness

**DOI:** 10.1186/s12966-020-01022-6

**Published:** 2020-09-18

**Authors:** Bojana Klepac Pogrmilovic, Andrea Ramirez Varela, Michael Pratt, Karen Milton, Adrian Bauman, Stuart J. H. Biddle, Zeljko Pedisic

**Affiliations:** 1 Institute for Health and Sport, Victoria University, Ballarat Road, Footscray, Melbourne, VIC 3001 Australia; 2 Mitchell Institute, Victoria University, 300 Queen Street, Melbourne, VIC 3000 Australia; 3 School of Medicine, Universidad de los Andes, Bogota, Colombia; 4 University of California San Diego Institute for Public Health, 9500 Gilman Drive, San Diego, USA; 5 Norwich Medical School, University of East Anglia, Norwich Research Park, Norwich, Norfolk NR4 7TJ UK; 6 Sydney School of Public Health, University of Sydney, Camperdown, Sydney, NSW Australia; 7 Centre for Health Research, University of Southern Queensland, 37 Sinnathamby Boulevard, Springfield Central, QLD 4300 Australia

**Keywords:** Physical activity, Sedentary behaviour, Global, Assessment, Audit, Policies

## Abstract

**Background:**

Evidence on current, national physical activity (PA) and sedentary behaviour (SB) policies is limited. We, therefore, analysed availability, comprehensiveness, implementation, and effectiveness of PA and SB policies internationally.

**Methods:**

In this cross-sectional study, Global Observatory for Physical Activity (GoPA!) Country Contacts from 173 countries were asked to provide data on their national PA and SB policies by completing GoPA! Policy Inventory. Data were collected for 76 countries (response rate = 44%).

**Results:**

Formal written policies for PA and SB were found in 92% (95% confidence interval [CI]: 86, 98) and 62% (95% CI: 50, 75) of countries, respectively. Sixty-two percent (95% CI: 51, 73) of countries have national PA guidelines, while 40% (95% CI: 29, 52) have SB guidelines. Fifty-two (95% CI: 40, 64) and 11% (95% CI: 3, 19) of countries have quantifiable national targets for PA and SB, respectively. The most represented ministries/departments involved in the promotion of more PA and/or less SB were in the sport (reported by 99% countries; 95% CI: 96, 100), health (97%; 95% CI: 94, 100), education (94%; 95% CI: 88, 100), and recreation and leisure (85%; 95% CI: 71, 99) sectors. The median score (0–10) for the comprehensiveness of PA and SB policies was 4 (95% CI: 4, 5) and 2 (95% CI: 2, 3), respectively. For PA and SB policy implementation it was 6 (95% CI: 5, 6). For the effectiveness of PA and SB policies it was 4 (95% CI: 3, 5) and 3 (95% CI: 2, 4), respectively. PA and SB policies were generally best developed in high-income countries and countries of European and Western-Pacific regions.

**Conclusions:**

Most of the included countries have PA policies, but their comprehensiveness, implementation, and effectiveness are generally low-to-moderate. SB policies are less available, comprehensive, implemented, and effective than PA policies. PA and SB policies are better developed in high-income countries, compared with low- and lower-middle-income countries, and in countries of European and Western-Pacific regions, compared with other world regions. More investment is needed in development and implementation of comprehensive and effective PA and SB policies, particularly in low- and lower-middle-income countries.

## Background

Insufficient physical activity (PA) and high sedentary behaviour (i.e. activities in sitting or reclining posture requiring low energy expenditure; SB) are jointly responsible for around 13% of deaths globally [1, 2]. Alongside smoking, unhealthy diet, and excessive alcohol consumption, insufficient PA and SB are key behavioural risk factors for the development of noncommunicable diseases [3, 4]. Insufficient PA is associated with a significant economic burden [5]. Its overall direct cost to worldwide healthcare systems is estimated to be around 53.8 billion international dollars [5]. Evidence on the considerable public health and economic benefits that could be achieved by increasing PA in the population has incentivised governments around the world to develop PA policies [6].

Research around PA policy is developing, and some data on PA policy are available for 168 countries [6]. SB policy research is a relatively new area [6], and for most countries evidence is lacking for the development of SB policies [6]. Research on national-level PA and SB policies may contribute to: (i) evidence-based development of new PA and SB policies; (ii) better implementation and evaluation of existing PA and SB policies; (iii) achieving sustainable reforms within the health, education, sport, and other sectors, particularly in regard to the promotion of more PA and less SB; (iv) raising awareness among policy makers and other public health stakeholders about existing challenges, gaps, and prospects in national-level PA promotion; (v) important debates between researchers and policymakers on existing and future PA and SB policies [7–16].

For the past several decades, national and subnational governments, international organisations such as the World Health Organization (WHO), public health researchers, and non-governmental organisations have worked on various initiatives to make the promotion of more PA and less SB a public health priority. In 2018, the WHO launched the *Global Action Plan on Physical Activity 2018–2030* urging countries around the world to implement policy actions that will support efforts to reduce levels of physical inactivity and SB and contribute to meeting the global target of a 15% relative reduction in the prevalence of insufficient PA by 2030 [17].

In 2012, the Global Observatory for Physical Activity (GoPA!) was established to monitor global progress in PA surveillance, research, and policy [18, 19]. The GoPA! is a council of the International Society for Physical Activity and Health [18, 19]. At the time when the GoPA! was established, little data on national PA surveillance, research, and policy were available that would allow for comparisons between different countries and world regions [18, 19]. In 2015, the GoPA! issued PA profiles for 139 countries, the so-called “PA Country Cards” [20]. The data presented in the Country Cards were a valuable starting point towards a better understanding of the global progress on PA policies [6]. The first set of Country Cards included information on research, surveillance and on the availability of national action plans for PA [20]. Including comprehensiveness, implementation, and effectiveness of PA policies as well as SB policy became one of the goals for the Second set of Country Cards to be released by the end of 2020. Furthermore, national policies change over time; hence, information on PA and SB policies needs to the regularly updated [6]. Therefore, the aim of this study was to audit and critically assess the availability, comprehensiveness, implementation, and effectiveness of current national-level PA and SB policies globally.

## Methods

### Data collection and study sample

The data collection in this cross-sectional study took place from October 2019 to March 2020. GoPA! Country Contacts from 173 countries were invited to participate in the study and provide information on national PA and SB policies in their countries. All GoPA! Country Contacts were invited to participate in the survey, regardless of whether their country had or did not have PA and SB policy. The GoPA! Country Contacts are an established group that were identified by the GoPA!: (i) using PubMed search of the PA literature; (ii) from the list of focal points of international networks for PA promotion; and (iii) from the list of focal points of the WHO regional offices. To be selected, Country Contacts needed to have established experience in the area of public health and PA as researchers, members of international networks for PA promotion or members of government institutions. More details about the selection of GoPA! Country Contacts can be found elsewhere [18, 20]. The *GoPA! Policy Inventory version 3.0* (Additional file 1), was distributed to the GoPA! Country Contacts as an online survey. Responses were obtained for a total of 76 countries (response rate = 44%), of which 51% were high-income, 28% upper-middle-income and 21% low and lower-middle-income. The study sample included countries from all six WHO regions. The most represented region was the European Region (38%), followed by the Region of Americas (22%), the African Region (12%), the Western Pacific Region (11%), the Eastern Mediterranean Region (11%), and the South-East Asia Region (5%). In 12 of the participating countries, we obtained separate responses from two Country Contacts. When their responses differed, we relied on the responses from the main Country Contact listed in the GoPA! Country Cards. Participation in the study was voluntary and all participants provided informed consent before responding to the survey questions. The study protocol was approved by the Victoria University Human Research Ethics Committee (ref: HRE19–057).

### Policy variables

In the *GoPA! Policy Inventory version 3.0,* we used a broad definition of PA policy, as recommended in the Comprehensive Analysis of Policy on Physical Activity (CAPPA) framework [21]. PA policy was “indicated by the totality of formal written policies, unwritten formal statements, written standards and guidelines, formal procedures, and informal policies (or lack thereof) that may directly or indirectly affect community- or population-level PA” [21]. Given the large overlap between the PA and SB policy fields, it is suggested that the CAPPA framework can also be used for the analysis of SB polices [21]. Therefore, we used the same broad definition from the CAPPA framework for SB policy*.*

The *GoPA! Policy Inventory version 3.0* contains 20 questions about national PA and SB policies. The questionnaire was developed based on: the Health enhancing physical activity policy audit tool, version 2.0 [22]; the monitoring framework from the European Union Recommendation on Health-Enhancing Physical Activity Across Sectors [23]; the CAPPA framework; and a year long process of engagement of stakeholders [21]. The questions on the *GoPA! Policy Inventory version 3.0* address the following elements of the CAPPA framework: *availability*; *formal written policies*; *written guidelines; formal procedures; actors*; *implementation;* and *effects* [21]. Specifically, the questions focus on: the availability of national formal written PA and SB polices (e.g., policy documents, legislation, strategies, action plans); national PA and SB guidelines; national targets for PA and SB; health surveillance or monitoring systems that include measures of PA and SB; ministries/departments involved in the promotion of more PA and less SB; and comprehensiveness, implementation and effectiveness of national PA and SB policies. When referring to the *availability of PA and SB policy*, we considered not only the availability of formal written PA and SB policies but also the availability of written guidelines, quantifiable targets, and national PA and SB surveillance or monitoring, because these are indicators of a government’s commitment or intention to support the promotion of more PA and less SB in the population [21]. The questions on comprehensiveness, implementation, and effectiveness of policies had ordinal response scales (0–10), with a higher value on the scale representing a better score. Detailed definitions of comprehensiveness, implementation and effectiveness of PA and SB policies are provided in Additional file 1.

### Data analysis

The data were analysed using IBM Statistical Package for the Social Sciences (SPSS), version 23 (SPSS Inc., an IBM Company, Chicago, IL, USA)*.* Ordinal data on comprehensiveness, implementation, and effectiveness of policy were presented using medians (and their 95% confidence intervals [CI]) and interquartile ranges. Categorical data were presented as percentages and their 95% confidence intervals. Data were analysed for the whole sample and stratified by WHO regions and country’s income level (GNI per capita, calculated using the Atlas method) according to the World Bank [24]. Differences in PA and SB policy between low-, middle, and high-income countries and between the WHO regions were analysed using the Kruskal-Wallis test, for ordinal variables, and chi-square test for categorical variables. The percentage of missing data was relatively low (range across variables: 0–9.2%, mean: 3.3%). In the analyses, we used pairwise deletion of missing data. We considered *p* < 0.05 as a threshold for statistical significance.

### Categorisation of countries

The list of 218 economies from June 2019 provided by the World Bank was used as the list of countries/states/economies [24]. The authors are mindful of the fact that some countries/states/economies on the World Bank’s list cannot be termed as “countries” because of unclear legal and/or political status. Nevertheless, for brevity purposes, we used the term “countries” as an abbreviation for “countries/states/economies”. In order to be consistent with previous analyses of national PA and SB policies globally, both by GoPA! [20] and other international organisations for PA promotion [25, 26], we separately analysed the four United Kingdom home nations; namely, England, Northern Ireland, Scotland, and Wales. The countries were divided into three groups by income level: high-income; upper-middle-income; and low and lower-middle-income, in accordance with the categorisation provided by the World Bank [24]. The two lowest income groups were merged into one, because of a small number of low-income countries in the sample. The countries were also categorised into the six WHO world regions: African Region; European Region; Eastern Mediterranean Region; Region of the Americas; South-East Asia Region; Western Pacific Region.

## Results

### Availability of PA and SB policies

#### Formal written PA and SB policies

We found that 92% (95% CI: 86, 98) of countries have national policy documents, legislation, strategies, or action plans that outline the government’s intention to increase PA. National policy documents, legislation, strategies or action plans that outline the government’s intention to tackle SB were found in 62% (95% CI: 50, 75) of countries. We found a total of 251 PA and SB policies. Sixty-eight per cent of all policies were published between 2015 and 2020.

**Fig. 1:**
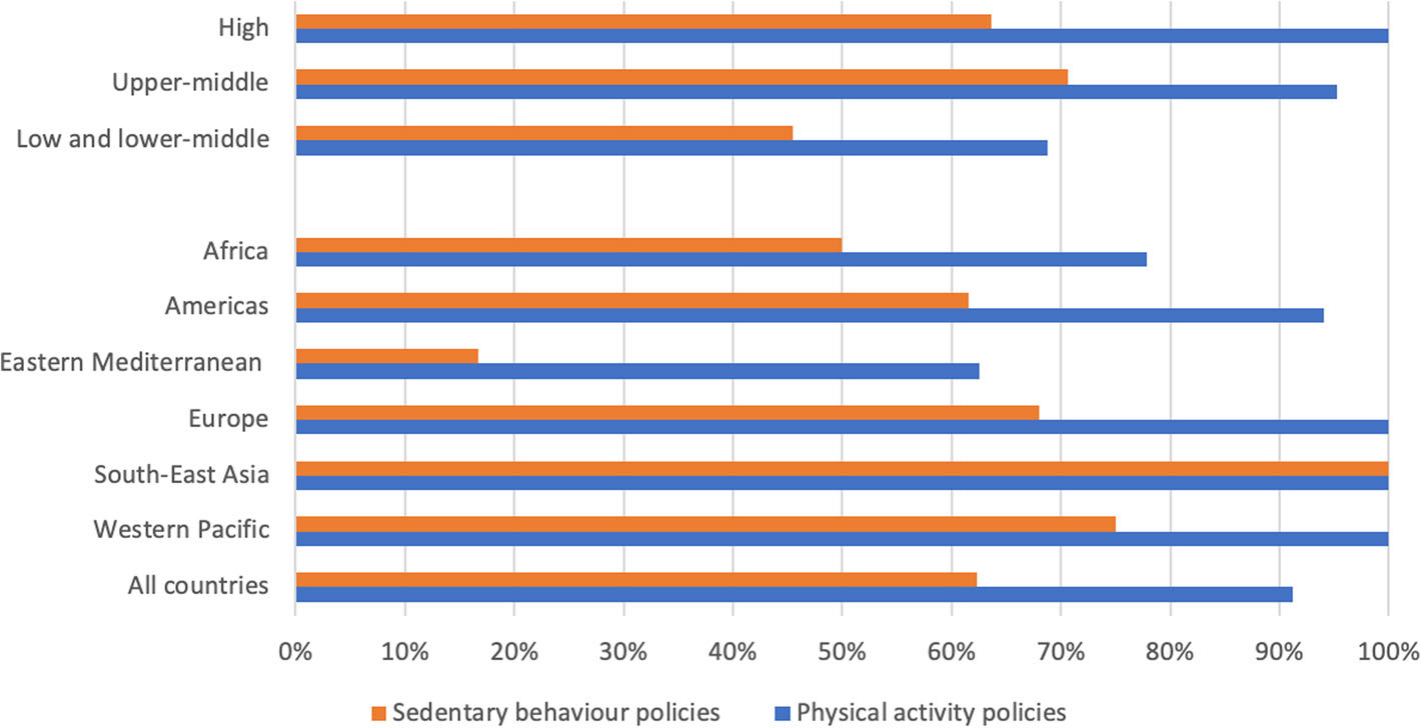
Percentage of countries with PA and SB policies, by income level and world region. PA: physical activity, SB: sedentary behaviour

The availability of national policies that aim to increase PA and tackle SB across different groups by income level and world regions is summarised in Fig. 1. We found significant differences in the availability of national policies to increase population PA between country groups by income level (*p* < 0.001) and between world regions (*p* = 0.007). We did not find a significant difference in the availability of national policies to tackle population SB by income level (*p* = 0.396) or by world region (*p* = 0.135).

#### PA and SB guidelines

**Fig. 2:**
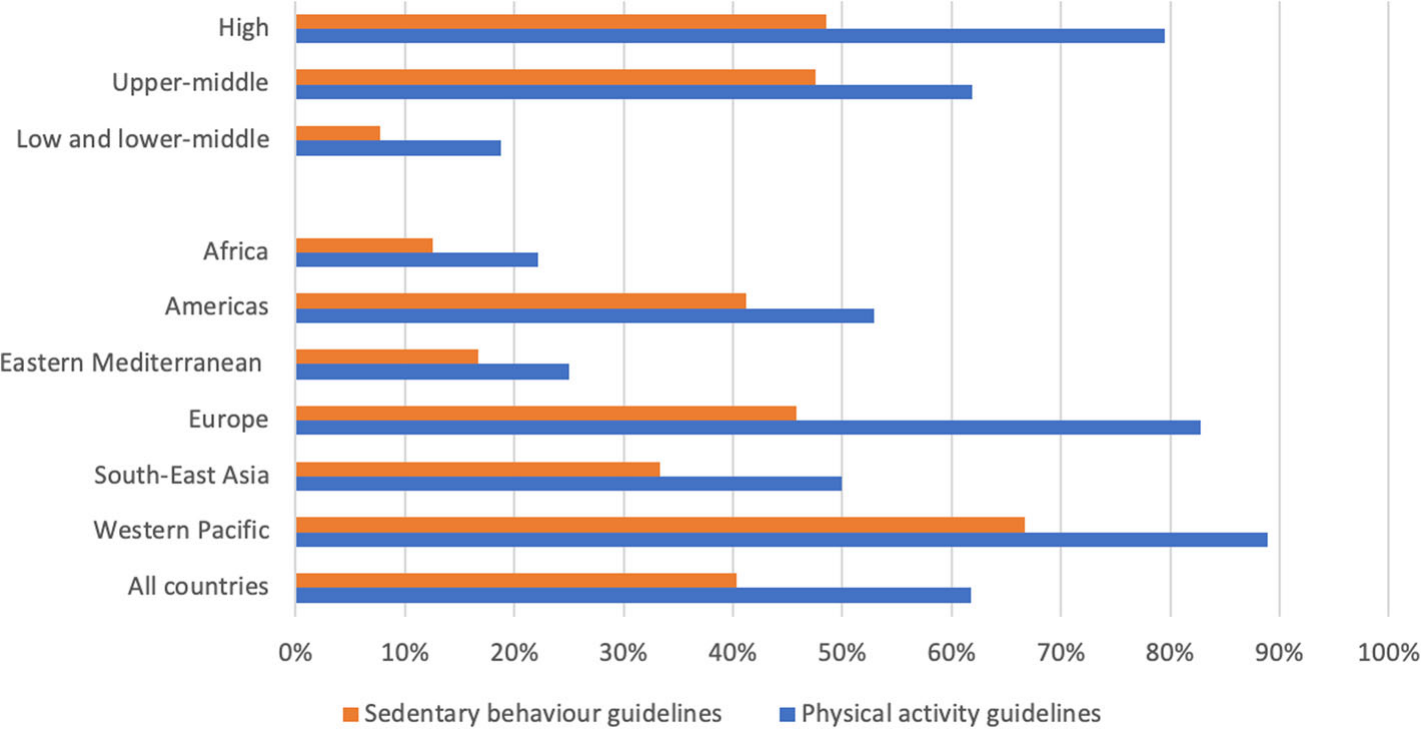
Percentage of countries with national PA and SB guidelines, by income level and world region. PA: physical activity, SB: sedentary behaviour

We found that 62% (95% CI: 51, 73) of countries have national PA guidelines, while 40% (95% CI: 29, 52) have guidelines for SB. The availability of national PA and SB guidelines across different income levels and world regions is summarised in Fig. 2. We found significant differences in the availability of PA guidelines between country groups by income level (*p* < 0.001) and between world regions (*p* = 0.002). We also found a significant difference in the availability of SB guidelines between country groups by income level (*p* = 0.028). We did not find significant differences in the availability of SB guidelines by world regions (*p* = 0.226).

**Fig. 3:**
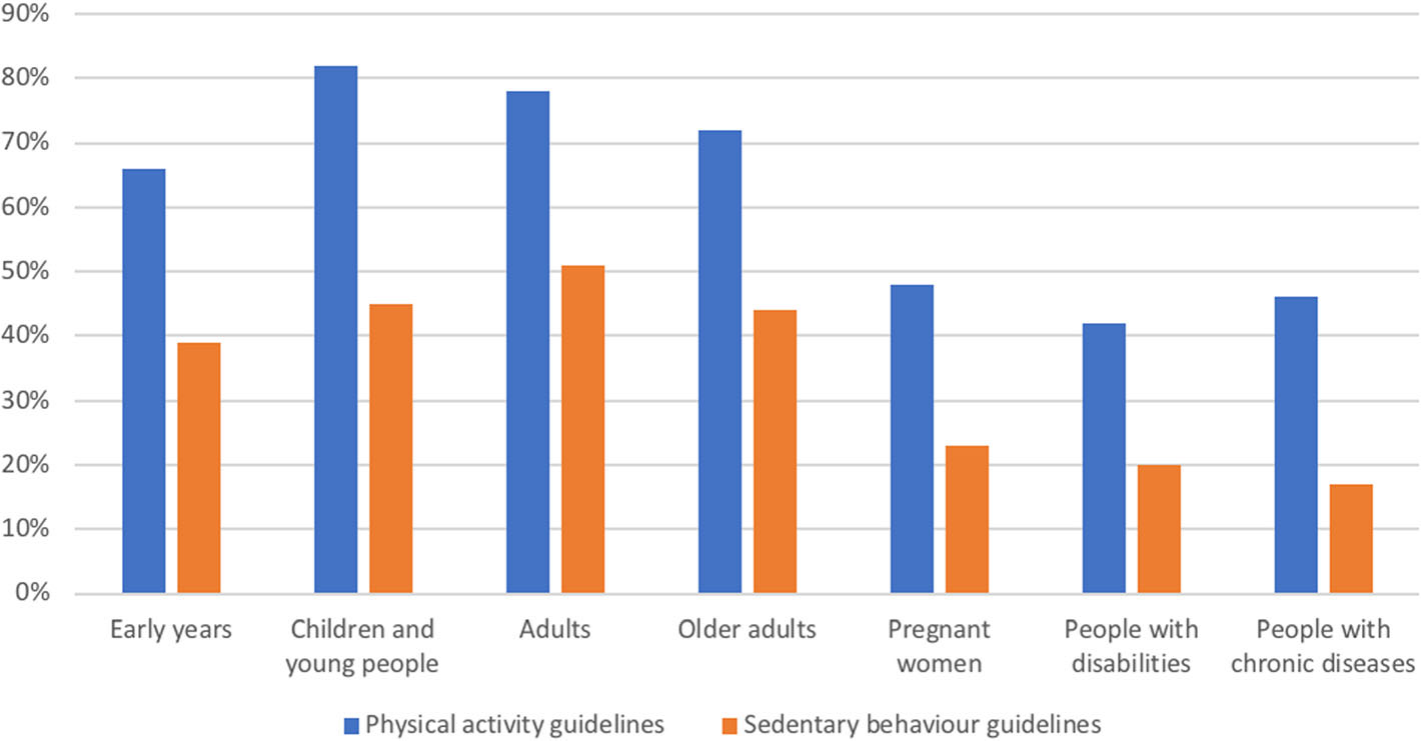
Percentage of countries with specific national PA and SB guidelines for different target groups. PA: physical activity, SB: sedentary behaviour

A large majority of countries have specific PA guidelines for *early years* (66%; 95% CI: 53, 79), *children and young people* (82%; 95% CI: 71, 92), *adults* (78%; 95% CI: 67, 89), and *older adults* (72%; 95% CI: 60, 84). About half of the countries have specific SB guidelines for *early years* (39%; 95% CI: 24, 54), *children and young people* (45%; 95% CI: 30, 60), *adults* (51%; 95% CI: 36, 67), and *older adults* (44%; 95% CI: 29, 59; Fig. 3). Specific national PA and SB guidelines for pregnant women, people with disabilities, and people with chronic disease were less well represented.

#### National targets for PA and SB

The availability of quantifiable national targets for PA and SB across countries with different income levels and world regions is presented in Additional file 2. Overall, 52% (95% CI: 40, 64) and 11% (95% CI: 3, 19) of countries reported having quantifiable national targets for PA and SB, respectively. We found significant differences in the availability of quantifiable national targets for PA between country groups by income level (*p* = 0.049) and between world regions (*p* = 0.027). We did not find significant difference in the availability of quantifiable national targets for SB by income level (*p* = 0.262) or by world region (*p* = 0.206).

#### National PA and SB surveillance/monitoring

The percentages of countries with national health surveillance or monitoring system that include measures of PA and SB, by income level and world regions, are presented in Additional file 3. Overall, 71% (95% CI: 60, 81) of countries have a national health surveillance or monitoring system that includes measures of PA, and 51% (95% CI: 39, 63) of countries have a national health surveillance or monitoring system with measures of SB. We did not find significant differences in the availability of national health surveillance/monitoring systems that include measures of PA and SB between countries with different income levels or between world regions.

### Ministries/departments involved in the promotion of more PA and less SB

The most represented ministries or departments with an active role in the promotion of more PA and/or less SB were in the sectors of: *sport* (reported by 99% of countries; 95% CI: 96, 100); *health* (97%; 95% CI: 94, 100); *education* (94%; 95% CI: 88, 100); *recreation and leisure* (85%; 95% CI: 71, 99); and *research* (68 95% CI: 26, 12). This was followed by the ministries or departments of *transport* (60%; 95% CI: 56, 74), *urban/rural planning and design* (60%; 95% CI: 45, 75), *tourism* (46%; 95% CI: 30, 62), *culture* (44%; 95% CI: 29, 59), *environment* (43%; 95% CI: 27, 58), *work and employment* (39%; 95% CI: 24, 54), and *public finance* (28%; 95% CI: 13, 42). The percentage of national ministries or departments involved in promotion of more PA and/or less SB are presented in Additional file 4.

### Comprehensiveness of PA and SB policies

**Fig. 4:**
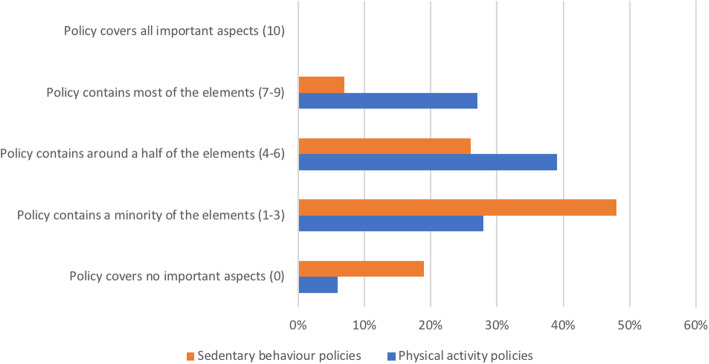
Distribution of national PA and SB policies according to their level of comprehensiveness. PA: physical activity, SB: sedentary behaviour

The distribution of national PA and SB policies according to their level of comprehensiveness is presented in Fig. 4. We found that PA policy in 39% (95% CI: 28, 51) of countries includes only around half of the important elements of a comprehensive approach (the list of elements can be found in Additional file 1), while in 27% (95% CI: 17, 37) of countries PA policy contains most of the important elements. A low level of comprehensiveness was found for PA policy in 28% (95% CI: 18, 39) of countries, while in 6% (95% CI: 0.3, 11) of countries PA policy covers no important elements. No countries reported having PA policy that includes all important elements. The median score for the comprehensiveness of PA policy was 4 (95% CI: 4, 5).

In most of the included countries, SB policy was assessed as having low comprehensiveness (48%; 95% CI: 35, 62) or as covering no important aspects (19%; 95% CI: 8, 29). Twenty-six per cent (95% CI: 14, 38) of countries reported having SB policy that includes only around half of important elements, while in 7% (95% CI: 0.4, 14) of countries SB policy contains most of the important elements. No countries reported having SB policy that includes all important elements. The median score for the comprehensiveness of SB policy was 2 (95% CI: 2, 3).

The level of comprehensiveness of PA and SB policies across countries with different income levels and world regions is presented in Table 1. We found significant differences in the comprehensiveness of PA policy between country groups by income level (*p* = 0.030) and between world regions (*p* = 0.049). We did not find significant differences in the comprehensiveness of SB policy by income level (*p* = 0.157) or by world region (*p* = 0.412). The level of comprehensiveness of PA and SB policies across different income levels and world regions is presented in Table 1.

**Table 1:** Level of comprehensiveness of national PA and SB policies, by income level and world region

Category	Physical activity policy			Sedentary behaviour policy		
	Median (IQR)	95% CI	*p*	Median (IQR)	95% CI	*p*
**Income**						
High	5 (3)	4, 7	0.030	2 (3.5)	1, 3.5	0.157
Upper-middle	4 (3)	3, 5		2.5 (3.25)	2, 4.5	
Low and lower-middle	2 (3.25)	1, 4		2 (2)	1, 3	
**Region**						
Africa	2.5 (3.75)	1, 5	0.049	2 (2)	1, 3	0.412
Americas	4 (3.75)	2, 5		2 (3.5)	1, 4	
Eastern Mediterranean	3 (5)	0, 5		1 (3.5)	0, 3.6	
Europe	5 (3)	4, 7		3 (3)	1, 4	
South-East Asia	6.5 (4.75)	2.7, 10		6 (4)	n/a	
Western Pacific	6 (5)	1, 8		2.5 (4.25)	0.2, 4.8	
**All countries**	4 (4)	4, 5	/	2 (3)	2, 3	/

*PA* Physical activity, *SB* Sedentary behaviour, *IQR* Interquartile range, *CI* Confidence interval for median, *p p*-value for the difference between groups from Kruskal-Wallis test, *n/a* number of countries too small to calculate CI

### Implementation of PA and SB policies

**Fig. 5:**
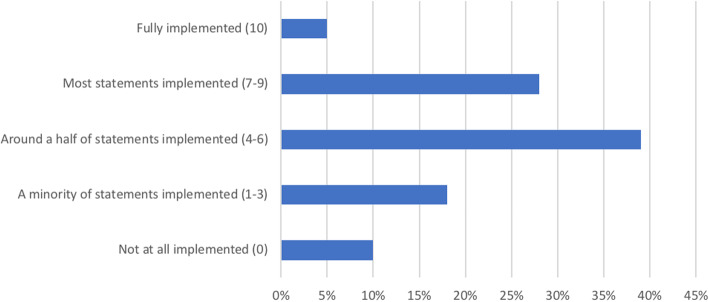
Distribution of PA and SB policies according to their level of implementation. PA: physical activity, SB: sedentary behaviour

The level of implementation was assessed for a total of 150 national PA and SB policies. The percentage of PA and SB policies according to their level of implementation is presented in Fig. 5. For 39% (95% CI: 27, 52) of policies, we found that only around a half of the statements were implemented, while for 28% (95% CI: 17, 39) of policies most statements were implemented. A low level of implementation was found for 18% (95% CI: 8, 28), while 10% (95% CI: 2, 17) of policies were not implemented at all. Only a few policies (5%; 95% CI: 0, 10) were fully implemented. The median score for PA and SB policy implementation was 6 (95% CI: 5, 6).

The level of implementation of PA and SB policies across countries with different income levels and world regions is presented in Table 2. We did not find a significant difference between the level of PA and SB policy implementation by income level (*p* = 0.059) or by world region (*p* = 0.166).

**Table 2:** Level of implementation of PA and SB policies, by income level and world region

Category	Median (IQR)	95% CI	*p*
**Income**			
High	6 (3)	5, 7	0.059
Upper-middle	6 (4)	3, 7	
Low and lower-middle	4 (5)	0, 5	
**Region**			
Africa	5 (6)	0, 6	0.166
Americas	6 (4.5)	3, 7.5	
Eastern Mediterranean	2 (6)	0, 6.2	
Europe	6 (2.75)	5, 7	
South-East Asia	6 (2)	n/a	
Western Pacific	6 (4)	3, 9	
**All countries**	6 (4)	5, 6	/

*PA* Physical activity, *SB* Sedentary behaviour, *IQR* Interquartile range, *CI* Confidence interval for median, *p p*-value for the difference between groups from Kruskal-Wallis test, *n/a* number of countries too small to calculate CI

### Effectiveness of PA and SB policies

**Fig. 6:**
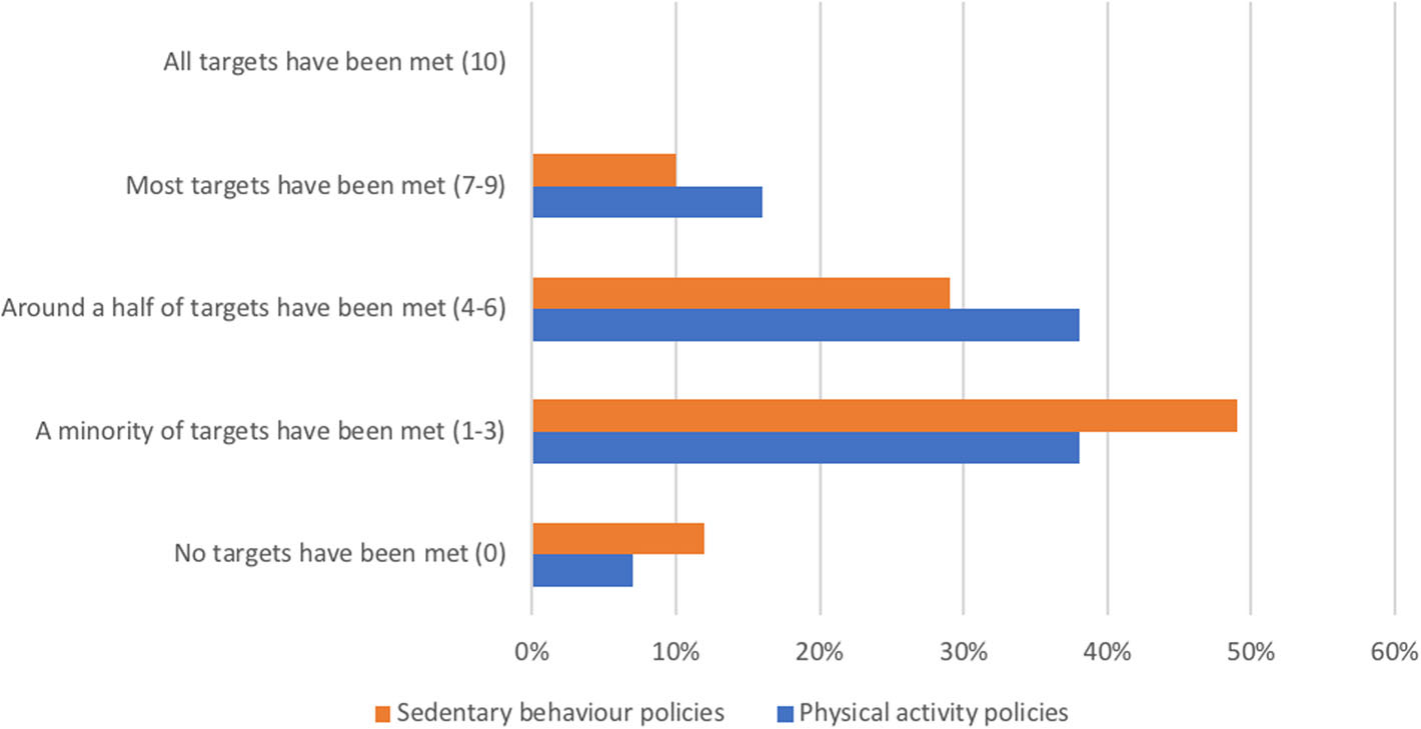
Distribution of PA and SB policies according to their level of effectiveness. PA: physical activity, SB: sedentary behaviour

The distribution of national PA and SB policies according to their level of effectiveness is presented in Fig. 6. We found that PA policy in 16% (95% CI: 7, 26) of countries was highly effective (i.e. most targets have been met), while in 38% (95% CI: 25, 51) of countries PA policy was moderately effective (i.e. around half of the targets have been met). A low level of effectiveness (i.e. a minority of targets have been met) was found for PA policy in 38% (95% CI: 25, 51) of countries, while in 7% (95% CI: 0.4, 14) of countries PA policy was not effective at all (i.e. no targets have been met). No countries reported having PA policy that was fully effective (i.e. all targets have been met). The median score for the effectiveness of PA policy was 4 (95% CI: 3, 5).

We found that SB policy in 10% (95% CI: 0.7, 19) of countries was highly effective (i.e. most targets have been met), while in 29% (95% CI: 15, 43) of countries SB policy was moderately effective (i.e. around half of the targets have been met). A low level of effectiveness (i.e. a minority of targets have been met) was found for SB policy in 49% (95% CI: 34, 64) of countries, while in 12% (95% CI: 2, 22) of countries SB policy was not effective at all (i.e. no targets have been met). No countries reported having SB policy that was fully effective (i.e. all targets have been met). The median score for the effectiveness of SB policy was 3 (95% CI: 2, 4).

The level of effectiveness of PA and SB policies across countries with different income levels and world regions is presented in Table 3. We found significant differences in the effectiveness of PA policy by income level (*p* = 0.004). We did not find significant differences in the effectiveness of PA policy by world regions (*p* = 0.175). We also did not find significant differences in the effectiveness of SB policy by income level (*p* = 0.202) or by world region (*p* = 0.265).

**Table 3:** Level of effectiveness of PA and SB policies, by income level and world region

Category	Physical activity policy			Sedentary behaviour policy		
	Median (IQR)	95% CI	*p*	Median (IQR)	95% CI	*p*
**Income**						
High	5 (3)	3, 5	0.004	3.5 (2.75)	3, 5	0.202
Upper-middle	5 (3)	3.5, 6		3 (3.25)	2, 5	
Low and lower-middle	3 (3.5)	0.5, 4		2 (3)	0, 3	
**Region**						
Africa	2 (4.25)	1, 6	0.175	2 (3)	1, 4	0.265
Americas	4 (3)	2, 5		3 (3)	1, 4	
Eastern Mediterranean	3 (3.5)	0.4, 5.6		2 (2)	n/a	
Europe	5 (4)	4, 6		5 (3)	3, 5	
South-East Asia	5 (2)	n/a		5 (2)	n/a	
Western Pacific	3 (4)	0, 6.2		3 (2)	n/a	
**All countries**	4 (2)	3, 5	/	3 (3.5)	2, 4	/

*PA* Physical activity, *SB* Sedentary behaviour, *IQR* Interquartile range, *CI* Confidence interval for median, *p p*-value for the difference between groups from Kruskal-Wallis test, *n/a* number of countries too small to calculate CI

## Discussion

In this international study conducted in 76 countries, we found that most of the included countries have formal written PA policies, guidelines for PA, health surveillance or monitoring systems that include measures of PA, and quantifiable national targets for PA. However, the levels of comprehensiveness, implementation and effectiveness of PA policies were generally found to be low-to-moderate. Compared with PA policies, national SB policies were generally less available and comprehensive. They were also less implemented and effective. PA and SB policies were generally more developed in high-income countries and countries of European and Western-Pacific regions.

### Availability of PA and SB policies

#### Formal written PA and SB policies

We found that formal written PA policies are available in most of the included countries, which is consistent with findings of previous studies [27, 28]. This is significant progress from the mid 2000s, when only around 29% of countries had PA policies [27]. However, our findings showed significant differences in the availability of national PA policies between country groups by income level and by world regions. The prevalence of insufficient physical activity is higher in high-income countries than in middle-income and low-income countries [29], which may partly explain why the governments in high-income countries are more likely to prioritise investing in the development of PA policies. Furthermore, in many low- and middle-income countries there is still a lack of country and context specific research on PA and health [30], which could be the reason for lower interest of policymakers to support the promotion of PA.

Low availability of formal written PA policies and PA guidelines may be especially problematic for the Eastern Mediterranean region. In addition to a high prevalence of noncommunicable diseases [31], this region has one of the highest physical inactivity and obesity rates in the world [32]. The call to focus more on developing national PA policies and implementation plans in the Eastern Mediterranean region from several years ago [33], is still justified.

The availability of SB policies was generally lower than the availability of PA policies. This finding is not surprising because public awareness of the potential adverse health outcomes of SB started to be systematically addressed no more than 20 years ago [6, 21]. Most evidence on SB policies and other determinants of SB comes from research conducted in high-income countries [6, 34]. Due to differences in socio-cultural, political, environmental, and legal factors, there is a need for context-specific research on SB policies [34]. More research on SB and associated policies is warranted, because such research may facilitate the development of national SB policies.

#### PA and SB guidelines

Availability of national PA guidelines is a good indicator of national PA and SB policy, as it shows the government’s intention to support the promotion of more PA and less SB. More effort needs to be put in the development of national SB guidelines, as they were less represented than PA guidelines. The low availability of SB guidelines might be because there is still an ongoing discussion within the research community on whether there is sufficient epidemiological evidence on the dose-response relationship between SB and health outcomes [35, 36]. Furthermore, we found that the difference between high-income and low- and lower-middle-income countries is particularly large in the availability of PA and SB guidelines. The fact that a large majority of low- and lower-middle-income countries do not have national PA and SB guidelines is concerning from a public health perspective. Greater investment is needed in the development or adoption of PA and SB guidelines in low- and lower-middle-income countries, to support their promotion of more PA and less SB in the population.

Most of the included countries have specific PA guidelines for early years, children and young people, adults, and older adults, in accordance with the target groups in the WHO PA recommendations [37, 38]. We found that national guidelines for other, specific target groups were much less represented. The guiding principle for the implementation of the *Global Action Plan on Physical Activity 2018–2030* is proportional universality, which states that greatest efforts should be directed towards target populations that are the least active [17]. Countries should consider adopting the proportional universality principle in the development and implementation of their national PA guidelines. In accordance with this principle, specific PA and SB guidelines should be developed for pregnant women, people with disabilities, and people with chronic disease, as these population groups tend to be less active and more sedentary than the rest of the population [39–41]. These will likely feature in the updated WHO guidelines, which might facilitate their adoption in countries [42]. It should be acknowledged that the development of specific recommendations for people with disabilities and chronic diseases may be challenging, due to a large variety of different disabilities and diseases and the fact that the guidelines may need to be disability/disease-specific. The research base supporting the development of specific recommendations for people with disabilities and chronic diseases is also less well developed.

#### National targets for PA and SB 

Health policy experts agree that for successful national PA and SB policies it is essential to set quantifiable, comparable national targets [22, 43–45]. However, we found that such targets for PA are still not available in nearly half of countries, while only a few countries have such targets for SB. The WHO’s “global” target of “a 15% relative reduction in the global prevalence of physical inactivity in adults and in adolescents by 2030” can only be achieved through the joint effort of all countries contributing to this common goal [17]. This target could be used as a basis for setting a national target for PA in a country that still does not have one, but it should be adapted to the country-specific context. Setting quantifiable targets for SB may be more challenging, because evidence on prevalence of SB and its trends is less developed.

#### National PA and SB surveillance/monitoring

Health surveillance and monitoring have a key role in assessing the progress towards meeting PA and SB targets [46, 47]. There are still a large number of countries that do not have PA surveillance, particularly in the Eastern Mediterranean region. We also found that national surveillance of SB is less common than PA surveillance. This suggests that many national governments are still not committed to systematically tracking PA and SB in the population, which means that they may not be able to assess their progress in relation to the WHO targets for 2030.

Previous studies have suggested that comprehensive PA and SB surveillance systems are needed to provide a good evidence base for public health interventions and strategies [46, 47]. Our study provided data only on availability of national PA and SB surveillance. Future studies should explore the comprehensiveness of PA and SB surveillance systems, and how they conform to the principles of optimal PA and SB surveillance [47].

### Ministries/departments involved in the promotion of more PA and less SB

An approach that integrates policies across settings and sectors is crucial for successful PA promotion at the national level [21, 44, 48–51]. We found that in most of the included countries ministries/departments in several sectors are, at least notionally, involved in the promotion of more PA and less SB, which suggests that, in this regard, national approaches to PA and SB policy are heading in the right direction. A PA policy audit conducted in several European countries suggested that the sport, health, and education sectors were key drivers of PA policy, and that more opportunities for PA promotion should be created in other sectors [14]. In addition to the ministries/departments of sport, health, and education, in most of the included countries we also found that ministries/departments of recreation and leisure, research, transport, and urban/rural planning and design are engaged in the promotion of more PA and less SB. Despite these encouraging findings, facilitating engagement of ministries/departments across different sectors in PA promotion remains an important task for national governments. There is still ample space for improvement, particularly in the tourism, culture, environment, work and employment, and public finance sectors. Ideally, whole-of-system [17] and structural approaches [52] would be applied, to engage all relevant sectors and utilise knowledge from public health and social sciences. As outlined in the *Global Action Plan on Physical Activity 2018–2030*, a whole-of-system approach may be necessary to enable adequate policy investments in PA [17].

### Comprehensiveness of PA and SB policies

Comprehensiveness is often regarded as a key determinant of successful policies on PA [49, 51, 53, 54]. Our findings suggest that in most of the included countries PA and SB policies are still not sufficiently comprehensive.

In 2013, a review of PA-related policies advocated for an urgent response to the noncommunicable disease burden in low- and middle-income countries by developing comprehensive policies to increase PA [55]. The results of our study show that the level of comprehensiveness of PA policies is higher in countries with higher income level. In our sample, the level of comprehensiveness of PA policies was the lowest in the African and Eastern Mediterranean regions. It may be challenging to develop all necessary components of PA and SB policy within the available budget, particularly in low- and lower-middle-income countries, where government’s spending on the prevention of non-communicable diseases is generally low, and where the prevention of infectious diseases is a competing priority [56, 57]. Limited funding should therefore be carefully distributed, to cover all the essential components of PA and SB policy. Low- and lower-middle-income countries and countries in the African and Eastern Mediterranean regions might benefit from greater support by international experts and organisations in the process of developing and refining their national PA and SB policies. Another option for some countries would be to consider implementing the WHO *Global Action Plan on Physical Activity 2018–2030* [17] and adapting their current PA policies accordingly. Governments, non-governmental organisations, academia, and other stakeholders involved in PA promotion are invited to align their efforts towards achieving the targets outlined in the plan [17].

### Implementation of PA and SB policies

A recent study found that most countries implemented less than a half of the noncommunicable disease policies recommended by the WHO [58]. The study also found that the number of countries that adopted PA policies is relatively large, but that it dropped between 2015 and 2017. We found that in most of the included countries half or more of the statements from key national PA and SB policies have not been implemented. Policies can be effective only if they are implemented; hence national governments should invest in mechanisms that would ensure better implementation of their PA and SB policies.

Several previous studies from high-income countries reported a lack of: (i) PA policy implementation; (ii) monitoring/evaluation of policy implementation; and (iii) allocated resources for PA policy implementation [25, 44, 49, 59]. From our data, it seems that the situation in low- and lower-middle-income countries is even more challenging, probably because they have fewer available resources for implementation of PA and SB policies. Highly complex policy designs without clear, specific, feasible, timely, and budgeted, and trackable action/implementation plans may be a recipe for failure of policy implementation [60, 61]. Therefore, national governments should rely on evidence from implementation science and aim to establish more efficient systems for implementation of PA and SB policies. National governments should also invest in rigorous evaluation of different types of interventions, sharing lessons learnt, and scaling-up the successful ones [62]. For some national governments, especially in low and lower-middle-income countries, PA promotion may not be a priority at the national level, so developing and piloting smaller-scale interventions at the local level could be a way to start building context-specific evidence.

### Effectiveness of PA and SB policies

Effective PA and SB policies are necessary to increase PA and reduce SB in the population. Previous studies reported a lack of evidence on the effectiveness of PA policy [25, 63]. Our findings indicate that the effectiveness of national PA and SB policies in most of the included countries is low to moderate. Timely modification of PA and SB policies is of utmost importance, if they prove to be ineffective. Although this may be a challenging task, countries should invest in establishing efficient and sustainable systems to evaluate national PA and SB policies, and use the gathered data to continuously improve the effectiveness of the policies.

### Strengths and limitations of the study

Strengths of this study include: (i) a large sample of countries from all world regions; (ii) separate analyses of PA and SB policies; and (iii) analyses of availability, comprehensiveness, implementation, and effectiveness of the policies.

This study was also subject to some limitations. First, not all the elements of a comprehensive analysis of PA and SB policy could be asked about, because we did not want to overburden our Country Contacts. For the same reason, we could not collect detailed data on all of the analysed policy elements. Second, the way policies are designed and implemented may vary depending on the political system, culture, and institutional settings in a given country [64]. Despite detailed explanations that we provided in our survey, it might be that some questions were not equally applicable to all country contexts. Third, the data were provided by GoPA! Country Contacts. It may be that some of them did not have access to all relevant data on PA and SB policies in their countries. Fourth, not all invited Country Contacts responded to the survey, which may have led to selection bias and reduced generalisability of the results. Finally, in the African and South-East Asian regions we had relatively small sample sizes, compared with other regions. This was mainly due to a lack of internationally visible PA and public health experts in some countries who we could recruit as Country Contacts.

## Conclusion

This study found that most of the included countries have formal written PA policies, guidelines for PA, quantifiable national targets for PA, and a health surveillance or monitoring system that includes measures of PA. However, the levels of comprehensiveness, implementation and effectiveness of these policies are generally low-to-moderate. Compared with PA policies, national SB policies are less available, comprehensive, implemented, and effective. Both PA and SB policies are more developed in high-income countries, compared with low- and lower-middle-income countries, and in countries of the European and Western-Pacific regions, compared with other world regions.

Future studies should aim to include more countries from the African and Eastern Mediterranean regions, and analyse elements of a comprehensive analysis of PA and SB policy [21] that were not covered in this study, such as country-specific policy contexts, political will, unwritten formal statements, and informal policies. The area would also benefit from a detailed analysis of all stages of the policy cycle and policies in specific sectors.

To conclude, the findings of this study indicate that more investment is needed in the development and implementation of comprehensive and effective PA and SB policies, particularly in low- and lower-middle-income countries.

## Supplementary information

**Additional file 1 ***GoPA! Policy Inventory, version 3.0*

**Additional file 2.** Percentage of countries with targets for PA and SB, by income level and world regions.

**Additional file 3.** Percentage of countries conducting PA and SB surveillance/monitoring, by income level and world region.

**Additional file 4.** Percentage of national ministries or departments involved in promotion of more PA and/or less SB.

## Data Availability

Summary results are available in Figures, Tables and Additional files. Raw data can be obtained from the corresponding author upon a reasonable request.

## Abbreviations

CAPPA: Comprehensive Analysis of Policy on Physical Activity
CI: Confidence Intervals
GoPA!: Global Observatory for Physical Activity
PA: Physical activity
SB: Sedentary behaviour
WHO: World Health Organization
