# Supplementary material for: National physical activity and sedentary behaviour policies in 76 countries: availability, comprehensiveness, implementation, and effectiveness
Source: Int J Behav Nutr Phys Act. 2020 Sep 18;17:116. doi: 10.1186/s12966-020-01022-6 (PMC7501705; doi:10.1186/s12966-020-01022-6)
Supplement: Supplementary file 1 — Additional file 1 GoPA! Policy Inventory, version 3.0 [file 12966_2020_1022_MOESM1_ESM.docx]

| **The Global Observatory for Physical Activity-GoPA! Policy Inventory**  Version 3.0, August 2019; Based on the Health-Enhancing Physical Activity Policy Audit Tool (HEPA-PAT, version 2.0),  the monitoring framework from the EU Recommendation on Health-Enhancing Physical Activity Across Sectors, and the  Comprehensive Analysis of Policy on Physical Activity (CAPPA) framework. |
| --- |

| This survey is conducted as a part of the second cycle of GoPA! Country Cards project. You have been invited to participate as a GoPA! Country Contact. Your participation in the survey is voluntary. You are not required to respond to all questions, and you may quit the survey at any time. However, it would be really important if you could respond to all survey questions. The survey will take about one hour to complete, depending on your responses.    Before you proceed, please read the [information for participants](http://docs.google.com/document/d/1sdb7vYDV-bGbeMOfAwx3PNFGpAMJbA0Y9vR5TnNB2ic/edit).    **Do you consent to participate in this survey?** | |
| --- | --- |
| Yes | No |
|  |  |

| A | **Your name and surname:** | |
| --- | --- | --- |
|  |  | |
| B | **Country for which you are providing responses:** | |
|  |  | |
| C | **Are you...** | |
|  | ... the main GoPA! Country Contact for this country? |  |
|  | ... a part of the GoPA! Country Card team for this country but not the main Country Contact? |  |
|  | … neither of the above (please write your position) |  |

| 1 | **Does your country have a physical activity plan/policy?** | | |
| --- | --- | --- | --- |
|  | Yes | No | Don’t know |
|  |  |  |  |

| 2 | **If yes, is it…** | | |
| --- | --- | --- | --- |
|  | … embedded in an NCD  prevention plan? | … standalone for physical activity? | Don’t know |
|  |  |  |  |

| 3 | **If your country has a physical activity plan/policy, please provide information about it.** | | |
| --- | --- | --- | --- |
|  | **Policy “A” title:** | Don’t know | N/A |
|  | Publication year: | Don’t know | N/A |
|  | Time frame covered: | Don’t know | N/A |
|  | Issuing body: | Don’t know | N/A |
|  | Web link (please also send a copy of the document by email to Andrea Ramirez Varela - Global Observatory for Physical Activity – GoPA! – Coordinator email; aravamd@gmail.com): | Don’t know | N/A |

| 4 | **Does your country have other current national policy documents, legislation, strategies, or action plans that outline the government’s intention to…** | | | |
| --- | --- | --- | --- | --- |
|  |  | Yes | No | Don’t know |
|  | … increase population physical activity? |  |  |  |
|  | … tackle population sedentary behaviour? |  |  |  |

| 5 | **If yes, please provide information about up to five most relevant ones.** | | |
| --- | --- | --- | --- |
|  | **Policy “B” title:** | Don’t know | N/A |
|  | Publication year: | Don’t know | N/A |
|  | Time frame covered: | Don’t know | N/A |
|  | Issuing body: | Don’t know | N/A |
|  | Web link: | Don’t know | N/A |
|  | **Policy “C” title:** | Don’t know | N/A |
|  | Publication year: | Don’t know | N/A |
|  | Time frame covered: | Don’t know | N/A |
|  | Issuing body: | Don’t know | N/A |
|  | Web link: | Don’t know | N/A |
|  | **Policy “D” title:** | Don’t know | N/A |
|  | Publication year: | Don’t know | N/A |
|  | Time frame covered: | Don’t know | N/A |
|  | Issuing body: | Don’t know | N/A |
|  | Web link: | Don’t know | N/A |
|  | **Policy “E” title:** | Don’t know | N/A |
|  | Publication year: | Don’t know | N/A |
|  | Time frame covered: | Don’t know | N/A |
|  | Issuing body: | Don’t know | N/A |
|  | Web link: | Don’t know | N/A |
|  | **Policy “F” title:** | Don’t know | N/A |
|  | Publication year: | Don’t know | N/A |
|  | Time frame covered: | Don’t know | N/A |
|  | Issuing body: | Don’t know | N/A |
|  | Web link: | Don’t know | N/A |

| 6 | Policy implementation includes translating statements, ideas, goals, and/or objectives mentioned in the policy documents into practice. For example, a policy document may mention building new facilities as one of the strategies to increase participation in physical activity. Implementation of this statement means having the new facilities actually built.    **Please estimate to what extent have the above-mentioned policies (described in questions 1 to 5 and marked by the letters A to F) been implemented.**    If the policy has been fully implemented, please grade its implementation as 10. Please grade the implementation of a policy from 7 to 9, if most of its statements have been implemented. Please grade the implementation of a policy from 4 to 6, if around a half of its statements have been implemented. Please grade the implementation of a policy from 1 to 3, if only a minority of its statements have been implemented. If the policy has not been implemented at all, please grade it as 0. | | | | | | | | | | | | | |
| --- | --- | --- | --- | --- | --- | --- | --- | --- | --- | --- | --- | --- | --- | --- |
|  | **Policy** | 0 | 1 | 2 | 3 | 4 | 5 | 6 | 7 | 8 | 9 | 10 | Don’t know | N/A |
|  | **“A”** |  |  |  |  |  |  |  |  |  |  |  |  |  |
|  | **“B”** |  |  |  |  |  |  |  |  |  |  |  |  |  |
|  | **“C”** |  |  |  |  |  |  |  |  |  |  |  |  |  |
|  | **“D”** |  |  |  |  |  |  |  |  |  |  |  |  |  |
|  | **“E”** |  |  |  |  |  |  |  |  |  |  |  |  |  |
|  | **“F”** |  |  |  |  |  |  |  |  |  |  |  |  |  |

| 7 | **Please provide the name of and/or link to the published sources (e.g. journal article, research document, technical report, thesis, dataset) that informed your answer. Please provide this information for each policy that you mentioned in your answers to the questions 1 to 5. Please write “my personal assessment” if your estimations were not informed by any other source.** | |
| --- | --- | --- |
|  | **Policy** | **Source(s)** |
|  | **“A”** |  |
|  | **“B”** |  |
|  | **“C”** |  |
|  | **“D”** |  |
|  | **“E”** |  |
|  | **“F”** |  |

| 8 | National recommendations are an official consensus statement issued by a governmental body and/or endorsed by the government. Physical activity recommendations typically state how much physical activity is required for health benefits, while sedentary behaviour recommendations typically suggest strategies for reducing prolonged periods of sitting.  **Does your country have national recommendations on…** | | | |
| --- | --- | --- | --- | --- |
|  |  | Yes | No | Don’t know |
|  | … physical activity? |  |  |  |
|  | … sedentary behaviour (i.e. sitting time)? |  |  |  |

| 9 | **If yes, do they include specific guidelines for…** | | | | | | |
| --- | --- | --- | --- | --- | --- | --- | --- |
|  |  | Physical activity | | | Sedentary behaviour | | |
|  |  | Yes | No | Don’t know | Yes | No | Don’t know |
|  | … early years (pre-school age)? |  |  |  |  |  |  |
|  | … children and young people (school age)? |  |  |  |  |  |  |
|  | … adults? |  |  |  |  |  |  |
|  | … older adults? |  |  |  |  |  |  |
|  | … pregnant women? |  |  |  |  |  |  |
|  | … people with disabilities? |  |  |  |  |  |  |
|  | … people with chronic diseases? |  |  |  |  |  |  |
|  | **Other target groups (please specify or write “no”):** | | | | | | |

| 10 | **If your country has national recommendations on physical activity and/or sedentary behaviour for children and young people, please provide information about them.** | | |
| --- | --- | --- | --- |
|  | **Physical activity recommendations** | | |
|  | Document name: | Don’t know | N/A |
|  | Publication year: | Don’t know | N/A |
|  | Recommended intensity(ies): | Don’t know | N/A |
|  | Recommended duration: | Don’t know | N/A |
|  | Recommended frequency: | Don’t know | N/A |
|  | Recommended duration of bouts: | Don’t know | N/A |
|  | Recommendation for additional benefits: | Don’t know | N/A |
|  | Recommended inclusion of vigorous activity: | Don’t know | N/A |
|  | Other recommendations: | Don’t know | N/A |
|  | **Sedentary behaviour recommendations** | | |
|  | Document name: | Don’t know | N/A |
|  | Publication year: | Don’t know | N/A |
|  | Recommendation for overall sitting: | Don’t know | N/A |
|  | Recommendation for screen time: | Don’t know | N/A |
|  | Other recommendations: | Don’t know | N/A |

| 11 | **If your country has national recommendations on physical activity and/or sedentary behaviour for adults, please provide information about them.** | | |
| --- | --- | --- | --- |
|  | **Physical activity recommendations** | | |
|  | Document name: | Don’t know | N/A |
|  | Publication year: | Don’t know | N/A |
|  | Recommended intensity(ies): | Don’t know | N/A |
|  | Recommended duration: | Don’t know | N/A |
|  | Recommended frequency: | Don’t know | N/A |
|  | Recommended duration of bouts: | Don’t know | N/A |
|  | Recommendation for additional benefits: | Don’t know | N/A |
|  | Muscle-strengthening activity recommendation: | Don’t know | N/A |
|  | Other recommendations: | Don’t know | N/A |
|  | **Sedentary behaviour recommendations** | | |
|  | Document name: | Don’t know | N/A |
|  | Publication year: | Don’t know | N/A |
|  | Recommendation for overall sitting: | Don’t know | N/A |
|  | Recommendation for screen time: | Don’t know | N/A |
|  | Other recommendations: | Don’t know | N/A |

| 12 | **If your country has national recommendations on physical activity and/or sedentary behaviour for older adults, please provide information about them.** | | |
| --- | --- | --- | --- |
|  | **Physical activity recommendations** | | |
|  | Document name: | Don’t know | N/A |
|  | Publication year: | Don’t know | N/A |
|  | Recommended intensity(ies): | Don’t know | N/A |
|  | Recommended duration: | Don’t know | N/A |
|  | Recommended frequency: | Don’t know | N/A |
|  | Recommended duration of bouts: | Don’t know | N/A |
|  | Recommendation for additional benefits: | Don’t know | N/A |
|  | Muscle-strengthening activity recommendation: | Don’t know | N/A |
|  | Balance and fall prevention recommendations: | Don’t know | N/A |
|  | Other recommendations: | Don’t know | N/A |
|  | **Sedentary behaviour recommendations** | | |
|  | Document name: | Don’t know | N/A |
|  | Publication year: | Don’t know | N/A |
|  | Recommendation for overall sitting: | Don’t know | N/A |
|  | Recommendation for screen time: | Don’t know | N/A |
|  | Other recommendations: | Don’t know | N/A |

| 13 | **Does your country have a national health surveillance or monitoring system that includes measures of…** | | | |
| --- | --- | --- | --- | --- |
|  |  | Yes | No | Don’t know |
|  | … physical activity? |  |  |  |
|  | … sedentary behaviour? |  |  |  |

| 14 | **Which ministries or departments in your national government have an active role in the promotion of more physical activity and/or less sedentary behaviour?** | | | | |
| --- | --- | --- | --- | --- | --- |
|  | **Ministry/department of:** | Yes | No | Don’t know | N/A: Please tick if your country does not have this ministry/department |
|  | Health |  |  |  |  |
|  | Sport |  |  |  |  |
|  | Recreation and leisure |  |  |  |  |
|  | Education |  |  |  |  |
|  | Transport |  |  |  |  |
|  | Environment |  |  |  |  |
|  | Urban/rural planning and design |  |  |  |  |
|  | Culture |  |  |  |  |
|  | Tourism |  |  |  |  |
|  | Public finance |  |  |  |  |
|  | Work and employment |  |  |  |  |
|  | Research |  |  |  |  |
|  | **Other ministries/departments (please specify or write “no”):** | | | | |

| 15 | **Does your country have quantifiable national targets (e.g. to increase the prevalence of meeting physical activity guidelines by 15% by 2030) for…** | | | |
| --- | --- | --- | --- | --- |
|  |  | Yes | No | Don’t know |
|  | … physical activity? |  |  |  |
|  | … sedentary behaviour? |  |  |  |

| 16 | **If yes, please provide information about up to five key targets, including their start and end years.** | | |
| --- | --- | --- | --- |
|  | **(1) Target:** | Don’t know | N/A |
|  | Time frame: | Don’t know | N/A |
|  | **(2) Target:** | Don’t know | N/A |
|  | Time frame: | Don’t know | N/A |
|  | **(3) Target:** | Don’t know | N/A |
|  | Time frame: | Don’t know | N/A |
|  | **(4) Target:** | Don’t know | N/A |
|  | Time frame: | Don’t know | N/A |
|  | **(5) Target:** | Don’t know | N/A |
|  | Time frame: | Don’t know | N/A |

| 17 | **Please estimate how comprehensive is your country’s current overall national policy (written, unwritten, or lack thereof) on physical activity and sedentary behaviour.**    Comprehensive policy uses multiple strategies for the promotion of more physical activity and less sedentary behaviour (e.g. individual-oriented behaviour change and environmental-focused interventions), covers multiple sectors and settings (e.g. health, sport, transport, and urban planning and design sectors), defines quantifiable targets, contains initiatives for specific population groups (e.g. children, people with chronic disease), includes a public awareness component (e.g. social marketing campaigns), defines the budget for implementation, includes multi-sectoral and/or cross-sectoral approaches, and defines policy evaluation strategies.    If the policy contains all of the elements, please grade its comprehensiveness as 10. If the policy contains most of the elements, please grade its comprehensiveness from 7 to 9. If the policy contains around half of the elements, please grade its comprehensiveness from 4 to 6. If the policy contains only a minority of the elements, please grade its comprehensiveness from 1 to 3. If it includes none of the elements, please grade it as 0.    **Please provide your rating for...** | | | | | | | | | | | | |
| --- | --- | --- | --- | --- | --- | --- | --- | --- | --- | --- | --- | --- | --- |
|  | … physical activity policy. | | | | | | | | | | | | |
|  | 0 | 1 | 2 | 3 | 4 | 5 | 6 | 7 | 8 | 9 | 10 | Don’t know | N/A |
|  |  |  |  |  |  |  |  |  |  |  |  |  |  |
|  | … sedentary behaviour policy. | | | | | | | | | | | | |
|  | 0 | 1 | 2 | 3 | 4 | 5 | 6 | 7 | 8 | 9 | 10 | Don’t know | N/A |
|  |  |  |  |  |  |  |  |  |  |  |  |  |  |

| 18 | **Please provide the name of and/or link to the source of evidence (e.g. evaluation report, journal article, research document, technical report, thesis) that informed your answer. Please write “my personal assessment”, if your estimation was not informed by any other source.** |
| --- | --- |
|  |  |

| 19 | Overall national policy is considered to be effective, if, as a result of it, the population’s physical activity levels have increased (or remained stable) or the population’s sedentary behaviour has decreased (or remained stable) as planned.  It is important to note that some policies may have a direct effect and others an indirect effect. For example, changes in the funding of the national transportation scheme to favour public transport may ultimately result in more utilitarian physical activity, such as walking to and from the tram stop. On the other hand, changes in local policies, such as opening school grounds for public use, may have more direct effects on physical activity levels.    One way to assess the effectiveness of overall policy is to determine whether the quantifiable targets outlined in the policy documents have been met. This information can usually be obtained from policy evaluation reports. If the effects of the national policy have not been formally evaluated (by a government or non-government agency), you may assess its effectiveness based on other available research that investigated the effects of policy on population physical activity/sedentary behaviour.    If all targets have been met, please grade the effectiveness of policy as 10. If most targets have been met, please grade the effectiveness of policy from 7 to 9. If around half of the targets have been met, please grade the effectiveness of policy from 4 to 6. If only a minority of the targets have been met, please grade the effectiveness of policy from 1 to 3. If no targets have been met, please grade the effectiveness of policy as 0.    **Please estimate how effective has your country’s current overall national policy been in…** | | | | | | | | | | | | |
| --- | --- | --- | --- | --- | --- | --- | --- | --- | --- | --- | --- | --- | --- |
|  | … increasing population physical activity. | | | | | | | | | | | | |
|  | 0 | 1 | 2 | 3 | 4 | 5 | 6 | 7 | 8 | 9 | 10 | Don’t know | N/A |
|  |  |  |  |  |  |  |  |  |  |  |  |  |  |
|  | … tackling population sedentary behaviour. | | | | | | | | | | | | |
|  | 0 | 1 | 2 | 3 | 4 | 5 | 6 | 7 | 8 | 9 | 10 | Don’t know | N/A |
|  |  |  |  |  |  |  |  |  |  |  |  |  |  |

**
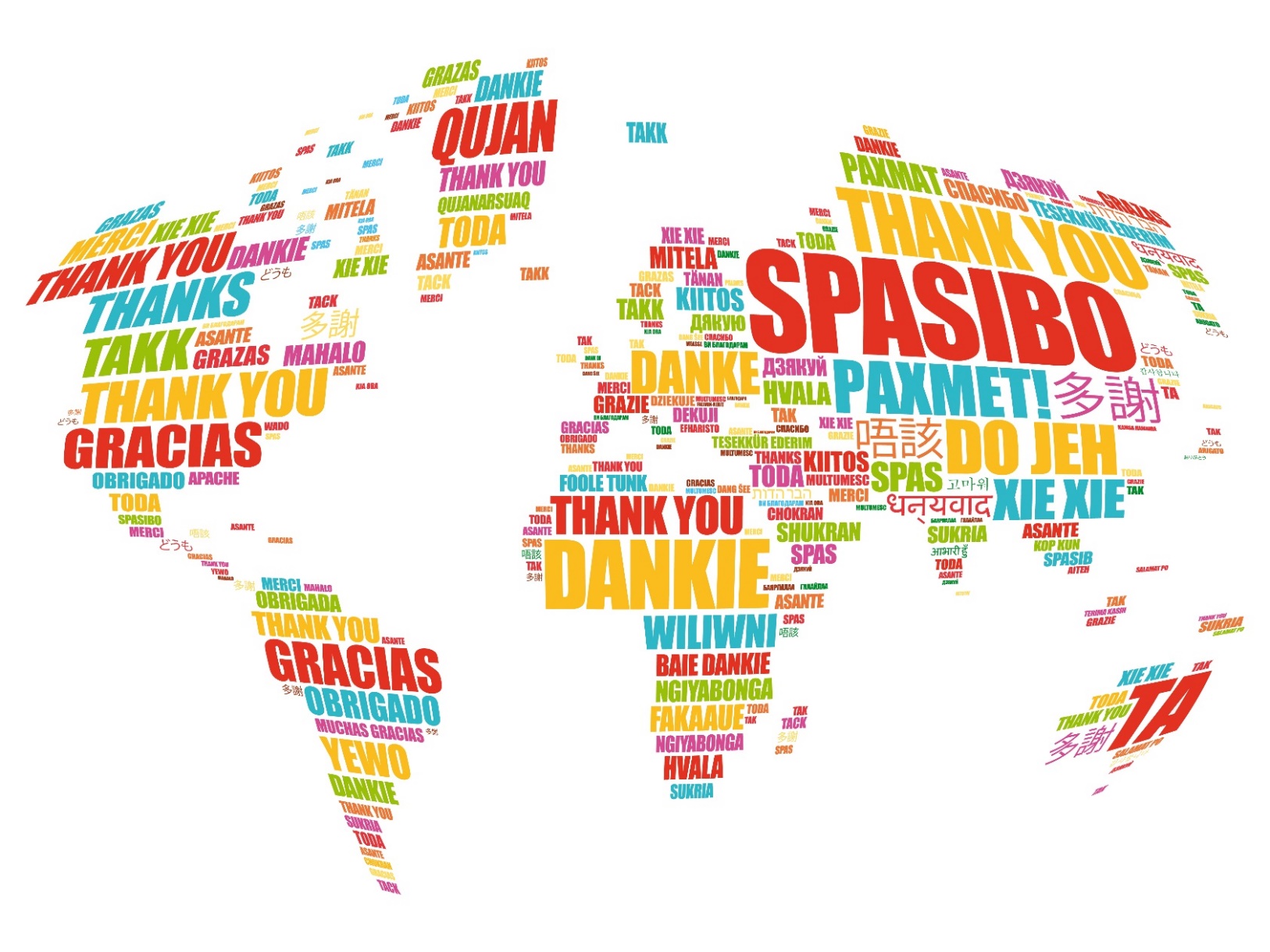
**

| 20 | **Please provide the name of and/or link to the source of evidence (e.g. evaluation report, journal article, research document, technical report, thesis, dataset) that informed your answer. Please write “my personal assessment”, if your estimation was not informed by any other source.** |
| --- | --- |
|  |  |
