# Supplementary material for: National physical activity and sedentary behaviour policies in 76 countries: availability, comprehensiveness, implementation, and effectiveness
Source: Int J Behav Nutr Phys Act. 2020 Sep 18;17:116. doi: 10.1186/s12966-020-01022-6 (PMC7501705; doi:10.1186/s12966-020-01022-6)
Supplement: Supplementary file 3 — Additional file 3. Percentage of countries conducting PA and SB surveillance/monitoring, by income level and world region. [file 12966_2020_1022_MOESM3_ESM.pdf]

Additional file 3 - Percentage of countries conducting PA and SB surveillance/monitoring, by income level and world region

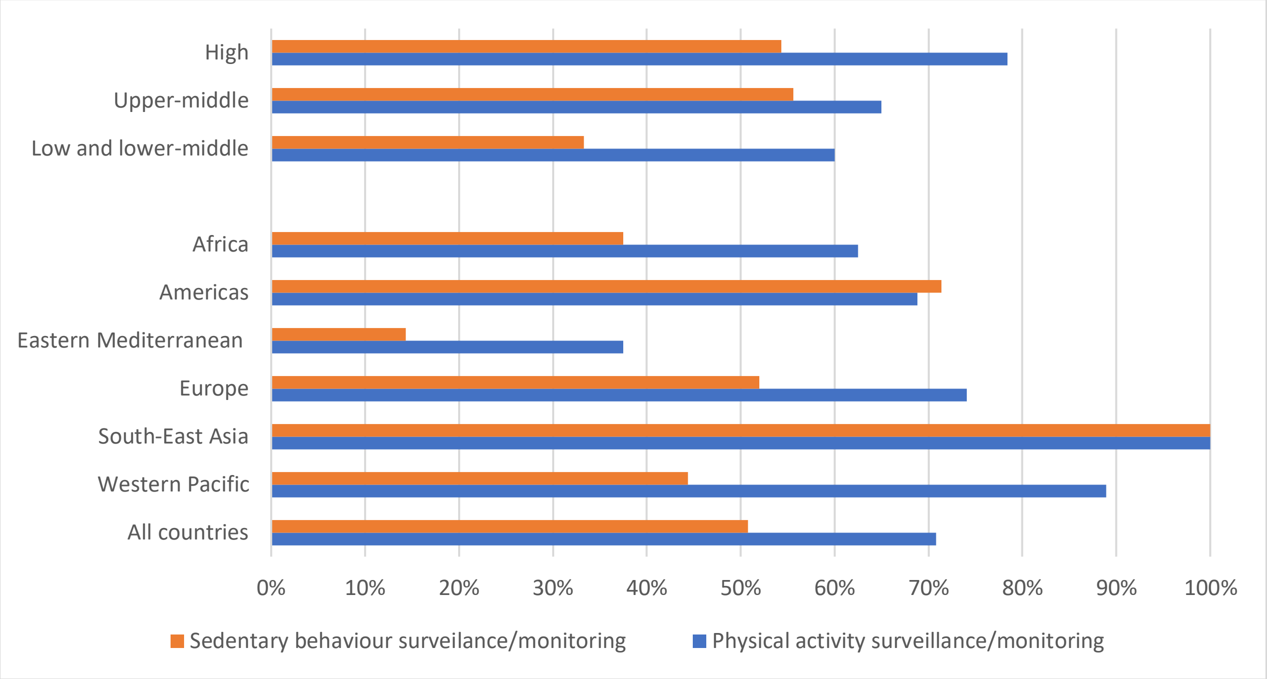

PA: physical activity, SB: sedentary behaviour
