# Supplementary material for: National physical activity and sedentary behaviour policies in 76 countries: availability, comprehensiveness, implementation, and effectiveness
Source: Int J Behav Nutr Phys Act. 2020 Sep 18;17:116. doi: 10.1186/s12966-020-01022-6 (PMC7501705; doi:10.1186/s12966-020-01022-6)
Supplement: Supplementary file 4 — Additional file 4. Percentage of national ministries or departments involved in promotion of more PA and/or less SB. [file 12966_2020_1022_MOESM4_ESM.pdf]

Additional file 4 - Percentage of national ministries or departments involved in promotion of more PA and/or less SB

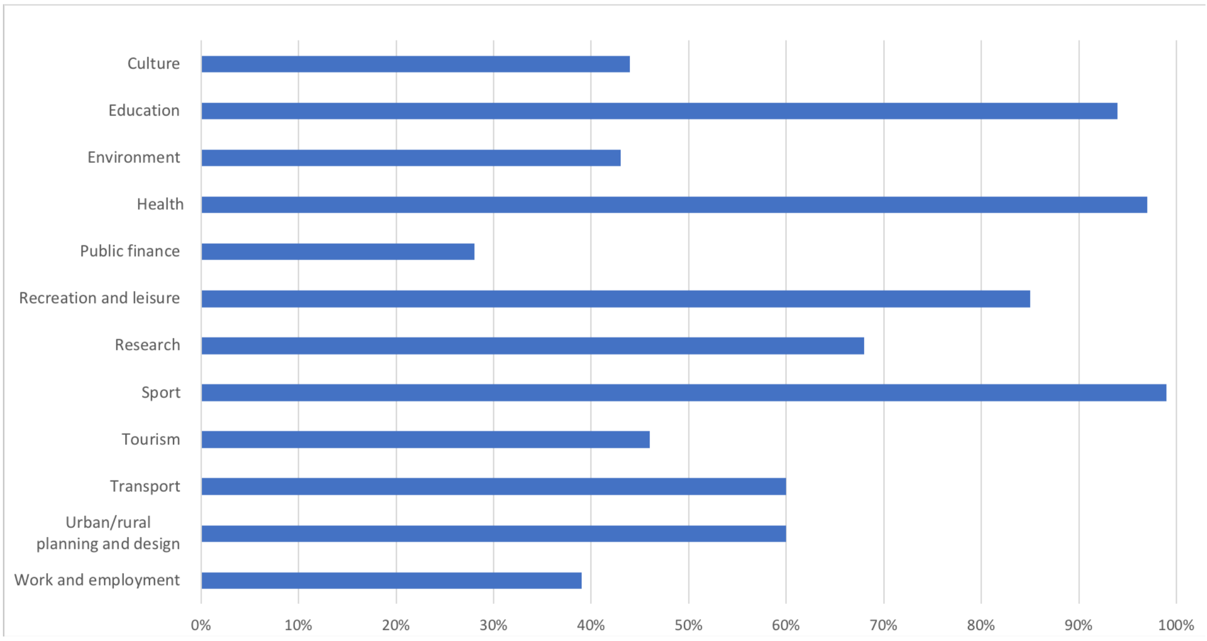

PA: physical activity, SB: sedentary behaviour
